# Socio-Demographic Determinants of Participation in Swimming Amongst Working Residents of Warsaw

**DOI:** 10.2478/v10078-012-0034-4

**Published:** 2012-05-30

**Authors:** Elżbieta Biernat

**Affiliations:** 1 Warsaw School of Economics, Warsaw, Poland.

**Keywords:** swimming activity, socio-demographic determinants, Warsaw

## Abstract

The aim of research is to assess the correlation between socio-demographic factors and swimming activity among the working population of Warsaw. The questionnaire survey included 4405 randomly selected residents of Warsaw. The correlation between the swimming activity and the variables characterizing the socio-demographic structure of the respondents were assessed by log-linear modelling. The significance of the impact of factors included in the analysis was determined using the chi-square test. Thirty-five per cent of the respondents declared recreational swimming. Gender, age, BMI, education, occupation, and income were significantly related to the swimming activity. Women (33%) – compared to men (38%) – were almost 1.2 times less likely to participate in swimming; similarly, overweight people (33%, OR = 0.90) and obese people (33%, OR = 0.92). People from Warsaw from 20–29 years (43%), with higher education (40%), incomes above the national average (40%), and representing the profession of an actor (52%), swam relatively more often. The results of the study might help in developing marketing plans and market segmentation strategies, as well as in forecasting the development trends of the leisure activity.

## Introduction

An active lifestyle is associated with a number of benefits important for society (Stephenson et al., 2000; Sallis & Owen, 1999) and health (WHO, 2002). However, despite extensive knowledge about the effectiveness of exercise in the prevention and treatment of diseases (Stephenson et al., 2000), 30% of the population of Europe (excluding Poles; Sjöström et al., 2006) and 28% of the Polish population (Central Statistical Office, 2009) could still be characterized by low levels of physical activity.

Regular physical activity of contemporary Poles is a developing process. On one hand, more and more people are engaged in regular physical activity, while on the other hand, for some social groups, this kind of activity is still unavailable (Biernat & Tomaszewski, 2011; Biernat et al., 2010). To obtain an explanation of the scale and nature of social disparities in this area, an analysis of the determinants of participation in active forms of leisure time is required. The active forms of leisure activities of various socio-professional groups are the subjects of many sociological studies. But when it comes to swimming, which is one of the most popular disciplines in Poland, studies mostly regard methods of teaching. They are mostly designed to fulfil sport-related objectives and described from the standpoint of the theory of sport. An analysis of the determinants of participation in this discipline is not that frequent. Therefore, the purpose of this study is to assess the correlation between demographic factors, such as sex, age, BMI, marital status, occupation, education, as well as income, and one of the most popular forms of physical education undertaken by respondents – swimming.

Respondents were randomly chosen mostly, from among professional groups that are internationally recognized as having social and economic prestige – the elite leadership groups that constitute a model for the rest of society. Thus, the result of this study might be helpful not only in building an effective strategy for sport development in the tested environment, but also might indirectly contribute to changes in the leisure time behaviour of society as a whole.

## Material and Methods

The study included 4405 randomly selected residents of Warsaw. The group was comprised of representatives of seven occupational groups: teachers (from high schools, middle schools, and primary schools), scientists (research institutes and academics), healthcare professionals, administrative personnel (central and local government employees), administrative and technical personnel (from universities, theatres, and research institution), trade industry professionals (hypermarkets, retail employees), and actors. The groups were distinguished according to International Standard Classification of Occupations (ISCO; www.ilo.org); however in the case of teachers, healthcare professionals, and actors originally representing the same category, it was decided to analyse these groups separately, as the representatives of these groups may be regarded as creators of the behaviour patterns and thus may influence behaviour of the general population (Cardinal, 2001; Cardon et al., 2009; Lobelo et al., 2009).

The study was conducted following the summer holiday season (November 2007–2008) and following the winter holiday season (March 2008–2009). In order to select the test group, a two-stage sampling system was used. The first step was to choose 3 to 10 institutions that employed people engaged in a particular profession from among all the institutions of that type in Warsaw. The exception was the group of retail employees, in which case three streets with a significant number of commercial buildings were selected in each district in Warsaw. In the second stage, a certain number of people in each institution were selected. At institutions employing up to 35 workers, the study embraced the whole group. In institutions employing or educating more people, a 30% sample group was selected, but limited to not more than 100 people.

The study was conducted in a survey method. Trained and supervised interviewers, according to a specific plan (the number of questions and their content were identical for all respondents) led direct interviews (standardized). The percentage of refusals to answer survey questions was small, and limited to a range of 3% to 5%. A slightly larger number of refusals were found in groups of trade industry professionals (10%) and actors (20%).

The poll – modified after the pilot version – included questions concerning the participation of respondents in recreational activities over the past year. The objects were asked about the nature of this participation (regular, periodic, sporadic), and type of sport disciplines or physical activities practiced recreationally, not professionally. The present paper is based on answers regarding the declaration of participation in swimming during the year. Besides the information concerning participation in swimming, interviewers collected data on the respondents’ gender, age, body weight and height, marital status, education, occupation, and income. Based on this data, the respondents were placed into various categories: age (20–29, 30–39, 40–49, 50–59, > 60 years old); BMI (underweight: < 20; standard: 20.0–24.9, overweight: 25.0–29.9; obesity: 30.0–39.9), marital status (single – never married women/man, divorced/widowed, in a stable relationship – marriage/partnership); education (primary/vocational, secondary, higher); income (< PLN 2500, > PLN 2500 gross per family member; based on data on website Money.pl (http://www.money.pl/) it is respectively approximately < €737 and > €737). The number of respondents in each category is shown in Table 1.

Correlations between the declarations regarding swimming and the variables that characterize the socio-demographic structure of respondents were evaluated based on a log-linear analysis. In order to determine the optimal model for testing, values of the chi-square test for main effects without interaction were calculated, and extended models were analysed considering higher-order interactions. The significance of the analysed correlations in the test model was evaluated on the basis of partial correlations and boundary correlations. A partial correlation indicates whether the given interaction affects the fitting of the model, when all other effects of the same order are already in the model. A boundary correlation allows for the comparison of the model without any interaction with the model, including only a given correlation. Hierarchical models were tested with different interactions. In order to fit the model with the observed values, an iterative procedure was also performed. The process was interrupted when the difference between the observed and fitted boundary distributions was not greater than the convergence criterion = 0.01. The results obtained in the analyses are presented using fractions, and odds ratios (OR) with 95% confidence intervals.

The analyses were performed using the statistical package of STATISTICA 8.0 PL. The significance level of p < 0.05 was assumed in assessing the significance of effects.

## Results

Among the working population of Warsaw, 35% declared swimming as an active form of leisure time. As a result of the analysis of partial and boundary correlations it was found that, among the analysed variables, only marital status showed no relation to the level of the respondents’ participation in swimming. Other variables, such as gender (partial χ^2^ = 11.8, p = 0.000), age (partial χ^2^ = 42.8, p = 0.000), BMI (partial χ^2^ = 10.3, p = 0.035), education (partial χ^2^ = 113.9, p = 0.000) and occupation (partial χ^2^ = 154.6, p = 0.000) were factors significantly associated with swimming. The same was true with regards to income (partial χ^2^ = 8.2, p = 0.017), which variable was included into the analysis despite a significant proportion of missing data (reaching 16%).

The analysis of boundary frequency tables estimated on the set models indicates that swimming was practiced relatively more often (p < 0.001) by men (38%) than by women (33%, Table 2). Women – compared with men – were almost 1.2 times less likely to practice this discipline.

The designated odds ratio for participation in swimming of underweight people (43%) was approximately 1.4 compared to those with normal BMI (35%). Contrast results were achieved for overweight people (33%, OR = 0.90) and people with obesity (33%, OR = 0.92).

In addition, swimming activity was more often undertaken (p = 0.000) by Warsaw’s young residents – aged 20–29 (43%), with higher education (40%) and level of income above the national average (40%). Thus, in comparison with the youngest respondents, the odds ratio of participation in swimming for people aged 30–39 years (37%), 40–49 years (32%), 50–59 years (30%) and > 60 years (27 %) was correspondingly reduced (OR = 0.79; 0.61; 0.57; 0.50). In turn, among the inhabitants of Warsaw with primary/vocational education (7%) and secondary education (26%) the risk of not practicing in this discipline was 8.3 and 1.9 times higher, respectively, than among those with higher education (40%). Respondents who declared income below the national average swam (34%, OR = 0.76) relatively less often (p = 0.017), then the respondents reporting above-average income.

It was also found, that there is correlation between the actual occupation and the practice of swimming. The observed interaction was largely reflected in differences between the actors (52%) and other groups: administrative staff (41%, OR = 0.65); healthcare professionals (41%, OR = 0.65); scientists (40%, OR = 0.63) and teachers (34%, OR = 0.49); administrative and technical staff (28%, OR = 0.37); and retail workers (18%, OR = 0.21).

## Discussion

Leisure time is an important component and inherent feature of contemporary social structures. As such, it is an object of sociological research in which behaviours of the various socio-professional groups are essential.

The studied respondents – working people in Warsaw (4405 individuals) – were recruited from seven professional groups (teachers, scientists, healthcare professionals, administrative personnel, administrative and technical stuff, trade industry professionals, and actors). These were mostly people with higher education (70%). Evaluated was their participation in swimming, a discipline that practiced rationally brings many health benefits (Barter, 1992; Parker, 2011), remedial and compensative benefits (Alavi et al., 2011; Jennings, 2011), as well as recreational benefits (Hastings et al., 1995).

On the basis of the self declaration, it was concluded that 35% of the respondents swam at least once a year. It seems that, in comparison with Canadians, among which 10.4% (5.5% of men and 18.7% women) practise this sport on a regular basis (at least once a week in the season or over a period of time during the year), it is not a satisfactory result (Ifedi, 2008). What then influences the level of participation of respondents in Warsaw in this discipline? Despite the fact that unmarried persons are considered to be a group that is very active in recreational activities (Lubowiecki-Vikuk, 2011), the analysis of the correlation of particular socio-demographic factors with swimming didn’t reveal a dependency regarding marital status. It has been shown, however, that gender age, BMI, education, occupation, and income are crucial variables that affect the behaviour of respondents. Previous publications showed a generally known relation that men are more active than women (ABS, 2003; Ifedi, 2008). Indeed, in this case, women are almost 1.2 times less like to participate in swimming. Henderson et al. (2000) explained the reasons for such a situation by the differences between men and women in the access and freedom of choice of recreation. This thesis was confirmed by Bittman and Wajcman (2000), who added an additional argument: namely the lower level of financial independence of women. It seemed that in Warsaw’s reality, major reasons for women lower participation in this discipline (33% vs. 38%) are on one hand – highlighted by Miller and Brown (2005) – family roles assigned to women; on the other hand, the – recently observed – distinctive increase of women’ professional activity. Consequently, work and family responsibilities significantly limit women's leisure time in Warsaw.

Swimming is often advertised as the best form of exercise for weight loss (Hastings et al., 1995). At the same time it is highlighted that it must be combined with rational nutrition. In the absence of diet, swimming has little or no effect on weight loss (Gwinup, 1987). Despite the high awareness of the respondents (17.3% of respondents comprised of healthcare professionals, 24.7% of teachers, 18.4% of scientists), the percentage of overweight (33%) and obese (33%) Varsovians who swim was not too high. Only underweight people are about 1.4 times more likely (compare to those with standard BMI) to practise swimming. It is difficult to definitively state the reason for such a situation. It doesn’t seem likely that among highly educated people lack of knowledge was an obstacle in this regard. Perhaps further studies on the correlation between motives, obstacles, frequency of swimming, and BMI of respondents would explain the problem.

The results also showed that age actually determines swimming activity among working resident of Warsaw. It is known that the risk of inactivity increases with age (Katzmarzyk, 2007). And in this case, when compared with the youngest respondents (aged 20–29 years), the risk for the 30–39-year-old group increased by 1.3 times; for 40–49-year-olds by 1.6 times; for the group of 50–59-year-olds by 1.8 times; and for people over 60 years old about 2 times. It is one of the global public health priorities to encourage older people into physical activity. It is a priority from the standpoint of physical health (Mensink et al., 1999), mental health (Mummery et al., 2004; Takkinen et al., 2001), and in terms of reducing mortality (Landi et al., 2004; Villeneuve et al., 1998). It seemed that swimming, as a form of sport that fulfilled all these requirements, would dominate the leisure time behaviour of the study participants. However, in this study, as well as in earlier studies conducted in Warsaw (Biernat et al., 2011), a downward trend has been noted in the older age groups. A similar problem was noticed among Canadians (Ifedi, 2008) and Swedes (Hagströmer et al., 2006). It seems that in this regard Australians should be the example to follow. Among them, more than half the population perform the recommended 30 minutes of physical activity almost every day (Armstrong et al., 2000).

Physical activity (including swimming) is not a simple function of age. In addition to many conditions, sport requires both some physical fitness and sufficient income. Respondents of this research were working (earning money), highly educated residents of Warsaw with particular cultural aspirations (Csikszentmihalyi, 1996). The correlation between level of education and care for one’s own physical condition seemed to be quite natural. Higher education entailed not only more knowledge on how to take care of one’s own health, but also the position in the occupational structure, which often requires creative approaches towards problems arising in the workplace. The high requirements related to occupation (care for appearance, slender body, and a high involvement in work, which must be combined with good physical fitness and resistance to stress) greatly contributed to the decision for regular fitness activity (Zarotis et al., 2007). It is not surprising, then, that a resident of Warsaw with higher education (40%) has more then an eight times greater chance of being involved in swimming, than those with a primary/vocational education (7%).

This statement is confirmed in virtually all studies, which show extremely high, positive levels of correlation not only between education and practicing different disciplines of sports (Ifedi, 2008), but also with the amount of the expenditure incurred for this purpose. However, Heilbrun and Gray (1993) believed that level of education has a stronger influence on recreational activity than level of income, yet many researchers highlight the fact that a lack of money can be a major obstacle, while higher incomes and mobility can be an important factor in undertaking physical activity (Ifedi, 2008; Merriman, 1991). Similarly, in the presented study, the financial situation of the participants highly influenced their participation in swimming. It turned out that people who reported the highest monthly income (above €737 gross per family member) practice swimming relatively more often (p = 0.000) than other respondents. With regard to income level, the obtained results should be interpreted with caution, as the percentage of a refusal to answer was significant (about 16% of the respondents).

The results also indicate that, although knowledge of the role of physical activity in maintaining health may be relevant in deciding whether to swim, among certain groups of respondents it is not a sufficient factor to continue such activity. The groups who are careless in this regard are easy to distinguish. It is unfortunate that, among those who do not swim – other than people that belong to the trade industry professionals group (82%) – are members from the group of healthcare professionals (59%). Compared to the group of actors – the group the most involved in swimming (52% of them swimming) – the chances of being engaged in swimming among the representatives of two aforementioned groups are, respectively, 4.8 and 1.5 times smaller. When analysing the impact of occupation on participation in swimming, it must be remembered that the classifications drawn up according to the performed occupation is based not only on the data regarding the amount and structure of free time or the leisure patterns of a particular occupation, but also based on the size of income – as demonstrated earlier in this paper.

At this point it is important to highlight that:

1. The chosen sample can be considered representative only for the working residents of Warsaw, with the exception of the blue-collar workers.
2. The results of the declared income and body mass should be interpreted with caution, as the percentage of refusal to answer was significant (16% and 7%, respectively). In this context, it is difficult to determine the credibility of the declarations of other respondents; that is to say, false declarations cannot be ruled out (resulting from a reluctance to disclose actual income or body weight). On the other hand (without ascribing a respondent’s intent to deceive), the real financial situation of the respondents can be stated based on data collected from over 4400 subjects.
3. No information on the motives and obstacles to undertake the activity of swimming, and a lack of information with regards to training frequency in specific environments, socio-professional groups, etc., significantly limited the ability to draw conclusions. Therefore, further study seems to be necessary to comprehensively explain the problem.

In conclusion, the findings of the research highlight the importance of the socio-demographic determinants of active forms of leisure time. It seems that the results of the study, including the strong correlations between factors such as gender, age, BMI, education, occupation, income, and swimming, might help in developing motivational programs and practical health-related activities conducted in the workplace. However, this requires further systematic research in various socio-occupational environments.

## Figures and Tables

**Table 1:** Number of subjects of the research (n=4405) within given category of socio-demographic variable

Variable	n	%
Sex		
Male	1533	34.8
Female	2872	65.2
Age		
20–29 years old	909	20.6
30–39 years old	1244	28.2
40–49 years old	991	22.5
50–59 years old	929	21.1
>60 years old	303	6.9
In a relationship		
No	1488	33.8
Yes	2881	65.4
BMI		
Underweight (<20)	482	10.9
Normal (20.0–24.9)	2272	51.6
Overweight (25.0–29.9)	1305	29.6
Obese (30.0–39.9)	287	6.5
Education		
Primary/Vocational	183	4.2
Secondary	1118	25.4
Higher	3098	70.3
Professional group		
Actors	103	2.3
Teachers	1089	24.7
Administrative personnel	627	14.2
Trade industry professionals	685	15.6
Scientists	810	18.4
Healthcare professionals	764	17.3
Administrative and technical staff	327	7.4
National average (737 €)^*^		
Below	3127	71.0
Above	589	13.4

* http://www.money.pl

Due to possible data deficiencies the number of respondents may vary between individual variables

**Table 2:** Factors determining swimming activity of employed residents of Warsaw (n=4405) and odds ratios (OR) as well as 95% confidence intervals (95% CI) established for being active

Variable	Swimming		Not-swimming		p	OR	95% CI
	
	n	%	n	%						
Sex					0.001		
Male	957	62.4	576	37.6		1	-
Female	1912	66.6	960	33.4		0.83	0.73–0.95
Age					0.000		
20–29 years old	518	57.0	391	43.0		1	-
30–39 years old	781	62.8	463	37.2		0.79	0.66–0.94
40–49 years old	679	68.5	312	31.5		0.61	0.50–0.73
50–59 years old	651	70.1	278	29.9		0.57	0.47–0.69
>60 years old	220	72.6	83	27.4		0.50	0.38–0.66
In a relationship					NS		
No	941	63.2	547	36.8			
Yes	1900	65.9	981	34.1			
BMI					0.035		
Underweight (<20)	276	57.3	206	42.7		1.39	1.14–1.70
Normal (20.0–24.9)	1478	65.1	794	34.9		1	-
Overweight (25.0–29.9)	880	67.4	425	32.6		0.90	0.78–1.04
Obese (30.0–39.9)	192	66.9	95	33.1		0.92	0.71–1.20
Education					0.000		
Higher	1862	60.1	1236	39.9		1	-
Secondary	832	74.4	286	25.6		0.52	0.44–0.60
Primary/Vocational	170	92.9	13	7.1		0.12	0.07–0.20
Professional group					0.000		
Actors	50	48.5	53	51.5		1	-
Administrative personnel	372	59.3	255	40.7		0.65	0.43–0.98
Healthcare professionals	452	59.2	312	40.8		0.65	0.43–0.98
Scientists	486	60.0	324	40.0		0.63	0.42–0.95
Teachers	715	65.7	374	34.3		0.49	0.33–0.74
Administrative and technical staff	235	71.9	92	28.1		0.37	0.23–0.58
Trade industry professionals	559	81.6	126	18.4		0.21	0.13–0.33
National average (737 €)^*^					0.017		
Below	2080	66.5	1047	33.5		0.76	0.64–0.92
Above	355	60.3	234	39.7		1	-

* http://www.money.pl

*NS – non-significant; Odds ratios (OR) were computed with reference to the not-swimming respondents*.

Due to possible data deficiencies the number of respondents may vary between individual variables
